# GPR Mapping of Cavities in Complex Scenarios with a Combined Time–Depth Conversion

**DOI:** 10.3390/s24103238

**Published:** 2024-05-20

**Authors:** Raffaele Persico, Ilaria Catapano, Giuseppe Esposito, Gianfranco Morelli, Gregory De Martino, Luigi Capozzoli

**Affiliations:** 1 Department of Environmental Engineering DIAM, University of Calabria, Via Pietro Bucci Cubo 44A, 87036 Rende, Italy; 2 Institute for Electromagnetic Sensing of the Environment-National Research Council of Italy (IREA-CNR), Via Diocleziano 328, 80124 Napoli, Italy; catapano.i@irea.cnr.it (I.C.); esposito.g@irea.cnr.it (G.E.); 3 Geostudi Astier srl, Via Edda Fagni 31, 57123 Livorno, Italy; gf.morelli70@gmail.com; 4 Institute of Methodologies for Environmental Analyses-National Research Council of Italy (IMAA-CNR), Contrada Loya, 85050 Tito Scalo, Italy; gregory.demartino@imaa.cnr.it (G.D.M.); luigi.capozzoli@imaa.cnr.it (L.C.)

**Keywords:** ground penetrating radar, cavity detection, combined time–depth conversion

## Abstract

The paper deals with a combined time–depth conversion strategy able to improve the reconstruction of voids embedded in an opaque medium, such as cavities, caves, empty hypogeal rooms, and similar targets. The combined time–depth conversion accounts for the propagation velocity of the electromagnetic waves both in free space and in the embedding medium, and it allows better imaging and interpretation of the underground scenario. To assess the strategy’s effectiveness, ground penetrating radar (GPR) data referred to as an experimental test in controlled conditions are accounted for and processed by two different approaches to achieve focused images of the scenario under test. The first approach is based on a classical migration algorithm, while the second one faces the imaging as a linear inverse scattering approach. The results corroborate that the combined time–depth conversion improves the imaging in both cases.

## 1. Introduction

Buried caves, voids, etc., represent potential safety problems [1,2]. On the other hand, they can also describe features of geological interest [3,4] or archaeological and cultural interest [5,6,7]. In some cases, cavities are underground secret paths or refuges exploited by criminals to hide arms, drugs, or just themselves [8]. In some cases, two or more of the exposed features are present together [9]. 

Whatever the practical use or technical issue, ground penetrating radar (GPR) imaging of a cavity is, in general, affected by a well-known compression effect that arises because the electromagnetic waves propagate with different velocities within the cavity and in the surrounding embedding medium. In particular, as well known, the waves are slower in the embedding medium depending on its relative permittivity $\epsilon_{r}$, linked in its turn to the material properties, i.e., its chemical composition, compactness, and theoretically also to its temperature and moisture content [10,11].

Theoretically, the incorrect GPR imaging of a cavity is related to the fact that approximate models are used to describe the scattering phenomenon to linearize the inverse problem at hand. However, mathematical (in particular in relationship with local minima) and computational difficulties make it difficult to address imaging as a nonlinear inverse problem [12,13,14]. This is especially valid in the framework of GPR prospecting, where the investigation domains are usually very large electrically, and the same cavities within the investigation can be sized hundreds or even thousands of times in the central internal wavelength. Consequently, classical migration algorithms [15,16] are commonly exploited for the focusing or possibly (essentially at a research level) more refined time reverse migration algorithms [17,18] or linear inverse scattering algorithms [19,20,21]. However, to our knowledge, none of these algorithms can avoid the above-quoted compression effect suffered by large cavities (in the order of tens or hundreds of central wavelengths). 

The combined time–depth conversion herein proposed here overcomes this o issue and allows for a correct estimation of the vertical size of a cavity to be achieved. However, this is possible only if both the ceiling and bottom of the cavity are visible in the data. This has been shown in [22] with a simulated Bscan. Herein, the experimental data and depth slices in particular will be shown.

A last point worth stressing is the problem of recognizing a cavity from GPR data. In fact, applying a combined time–depth conversion to a target only makes sense if this is a cavity and such information is not known a priori, except in the case of controlled data. Currently, there is no mathematical method that can verify a detected target as a cavity. However, some features of the data can suggest the presence of a cavity with high probability. In particular, hints are given by the context—the presence of strong reflection with inverse polarities from the top and from the bottom of the target, and a possible “X-shaped” signature due to the (vertical) lateral walls [5,6]. In some cases, an endoscopic sound with a small local carrot can reveal the presence of a shallow underground void cheaply and easily [23].

The combined time–depth conversion procedure is explained in the next section. In Section 3, the experimental testbed and performed case study are illustrated. In Section 4, the applied processing algorithms are described. Section 5 shows the results achieved both from a migration-based and inverse scattering-based algorithms; the conclusions follow.

## 2. The Combined Time–Depth Conversion (CTDC)

**Figure 1:**
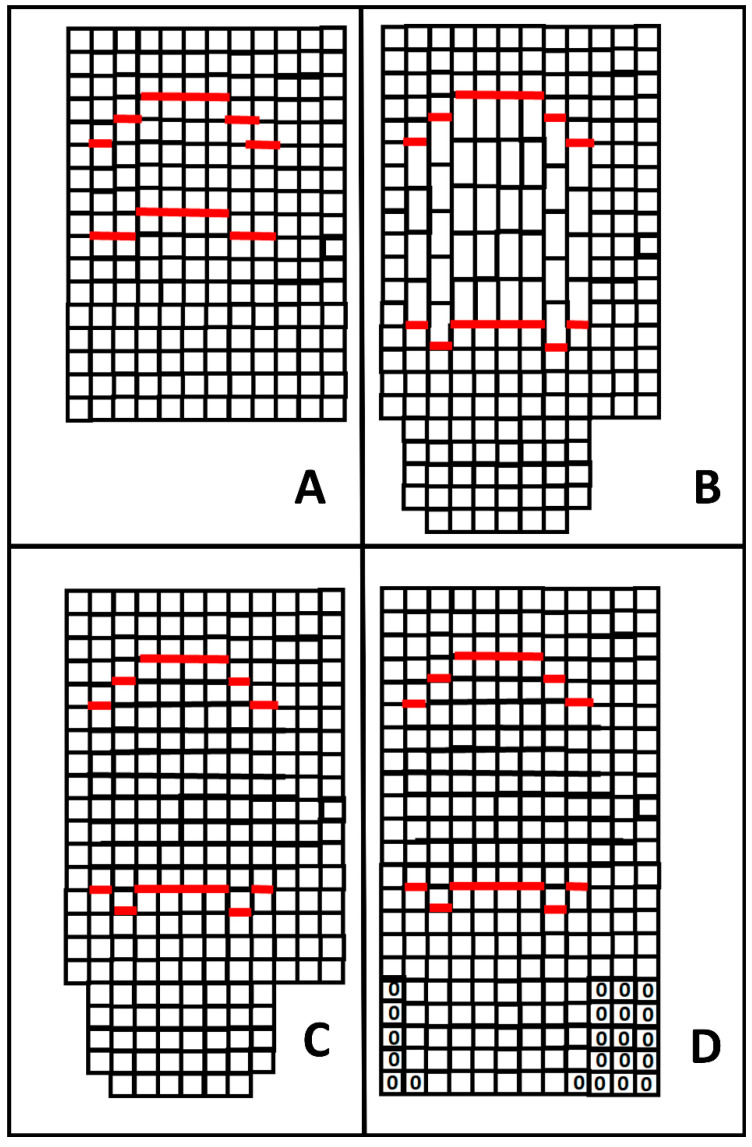
(**A**) Reconstruction of a cavity in abscissa and time. (**B**) Time–depth conversion of the cells according to the local propagation velocity. (**C**) Resampling of the values within the cavity. (**D**) Final zero padding.

This section presents the rationale of the combined time–depth conversion (CTDC) for cavities, which is schematically represented in Figure 1. The input is the focused image of a cavity in the abscissa and time domain. The achievement of such an image from GPR data is not a troublesome issue because a common migration algorithm usually achieves a good result in this sense [23]. So, let us start from the sketch of Figure 1A. The image represents a focused image of a cavity in the *x-t* plane (abscissa and time). The top of the cavity is not necessarily flat, nor is the bottom. This is common, especially in the case of natural cavities. On the other hand, even if the floor of the cavity is flat in the spatial domain, generally, it does not appear to be perfectly flat in the time domain due to the curvature of the ceiling. 

Let us now label as c the propagation velocity of the electromagnetic waves in the soil. The vertical size of the cell in the soil in Figure 1A is given by the time step Δt set by the human operator at the data collection stage. However, the corresponding spatial step in the soil is provided by:

(1) $$\Delta z_{1}=\frac{c\Delta t}{2}$$

whereas within the cavity, the spatial step should be given instead by:

(2) $$\Delta z_{2}=\frac{c_{O}\Delta t}{2}$$

where $c_{O}\approx 30\;cm/ns$ is the propagation velocity of the electromagnetic in air. 

Figure 1B depicts the vertical size associated with the pixels enclosed in the cavity according to this correspondence. Of course, the pixels into the cavity are vertically longer than those outside because the electromagnetic waves propagate with a faster velocity in the air than in the embedding medium. 

From Equations (1) and (2), it is also immediately retrieved that:

(3) $$\Delta z_{2}=\frac{c_{O}}{c}\Delta z_{1}$$

Namely, the ratio between the two “natural” depth steps inside and outside the cavity is given by the ratio between the propagation velocities in the cavity and in the embedding medium. It is worth pointing out it is not necessary that $\Delta z_{2}$ is an integer multiple of $\Delta z_{1}$: in Figure 1B, this occurs for mere drawing simplicity. On the other hand, the GPR result has to be represented as a matrix, which implicitly accounts for pixels of the same size. To satisfy such a constraint, the most reasonable choice is preserving the size of the “shorter” pixels in Figure 1B, which is done by resampling the longer ones (i.e., the pixels into the cavity). In detail, to perform the resampling correctly, one has to estimate the time–depth extension of the cavity, and this is done in Figure 1A by picking up columns per column from the top and the bottom of the cavity. Labeling $T_{top}$ as the time depth of the top and $T_{bot}$ as the time depth of the bottom, the thickness of the cavity in the time domain is estimated as:

(4) $${\Delta T=T}_{top}-T_{bot}$$

and its corresponding spatial thickness Δz is achieved by:

(5) $$\Delta Z=\frac{c_{O}\Delta T}{2}$$

Moreover, let N be the number of pixels corresponding to the cavity in the time domain, N is equal to:

(6) $$N=\frac{\Delta T}{\Delta t}$$

Since we want to sample the distance ΔZ with a spatial step $\Delta z_{1}$, it is easily demonstrated that the N samples within the cavity have to be resampled along $N_{1}$ values, where $N_{1}$ is given by:

(7) $$N_{1}=N\frac{c_{o}}{c}$$

In fact, we have:

(8) $${\Delta z_{1}N}_{1}=\frac{c\Delta t}{2}N\frac{c_{o}}{c}=\frac{\Delta T}{\Delta t}\frac{\Delta tc_{o}}{2}=\frac{\Delta Tc_{o}}{2}=\Delta Z$$

In general, the ratio between the velocities is not an integer number, and this means that the quantity calculated in Equation (7) is to be meant as the integer number closest to the right member of the equation. This means that a residual distortion remains. However, it is of the order of $\Delta z_{1}$ and is usually negligible. Figure 1C shows the interpolation step. Note that the entity of the resampling depends on the abscissa because, as said, the ceiling and the floor of the cavity, in general, are not flat. Consequently, the value of $N_{1}$ is calculated at each abscissa where the cavity is present, which means that both the top and the bottom of the cavity have to be detected in the *x-t* domain. However, after the interpolation, an incomplete matrix is achieved (see Figure 1C). Physically, this is coherent with the fact that the spatial maximum depth investigated is not constant vs. the abscissa, because the time bottom scale is constant vs. the abscissa. Still, the average velocity met along any vertical path changes. A simple way to complete the matrix is a zero padding, as illustrated in Figure 1D. In practice, the zero padding can be made invisible by cutting the image before its end and pre-adopting a redundant time bottom scale so that the bottom of the image will be essentially composed of noise.

As shown in Figure 1, the combined time–depth conversion modifies the shape of the cavity with respect to the surrounding scenario. Indeed, this also reflects the reciprocal positions of possible targets beyond the cavity, as we will show through the reported experimental tests. 

## 3. Experimental Tests

Experimental GPR data refer to a test site prepared at the Hydrogeosite Laboratory of the Institute of Methodologies for Environmental Analyses of the National Research Council of Italy (IMAA-CNR), Marsico Nuovo, Potenza, Italy. Specifically, an empty box plus three metallic pipes were displaced in a pool filled with silica sand. The empty box simulated the cavity and was buried with its top at a depth level of 35 cm. The box size was 80 × 45 cm, and its thickness was 40 cm. Then, three pipes, about 100 cm long, were displaced from left to right at a depth of 60 cm (on the left of the box), 40 cm, and 30 cm (on the right of the box).

**Figure 2:**
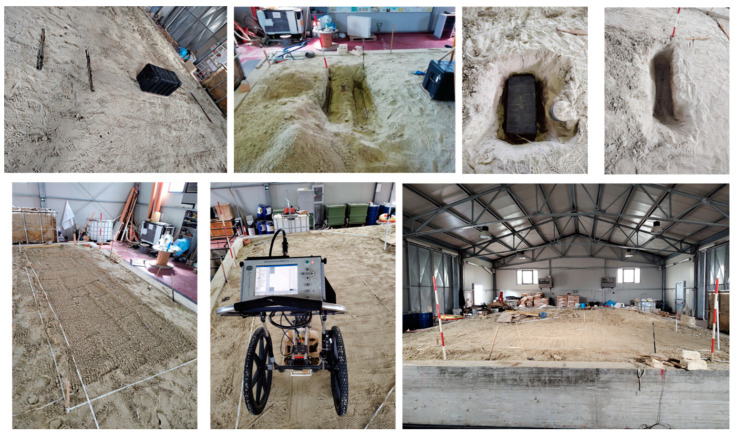
Set-up for the measurements with phases of the excavation for displacing the targets.

**Figure 3:**
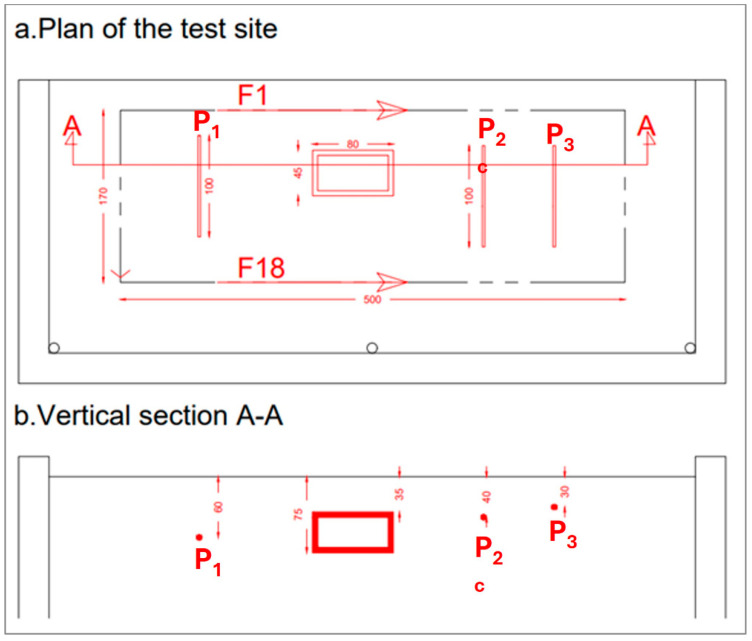
Map (upper panel) and cross-view (lower panel) of the buried targets that are a parallelepiped cavity plus three metallic pipes labeled as P_1_, P_2_, and P_3_. The path of the first Bscan (F_1_) and the last (F_18_) are also shown. The units are in centimeters.

Figure 2 shows the test bed during the displacement of the targets, whereas Figure 3 shows a quantitative sketch of the buried targets. 

After the target displacement, the whole site was flooded and left to dry to minimize the excavation’s track and make the targets embedded in homogeneous sand.

A GSSI SIR-3000 system (GSSI, Nashua, NH, USA) equipped with an antenna working at the central frequency of 900 MHz was exploited to gather the data. The in-line spatial step of the data was 1 cm, whereas the time step was 0.0488 ns. Data were collected along 18 measurement lines (or B-scans) parallel to each other and with interline space (transect) equal to 10 cm. The Bscans were about 4.85 m long, with some small variation from line to line, and 512-time samples were set for each GPR trace (A-scan). 

## 4. Data Processing

Before performing the combined time–depth conversion, two data processing approaches were applied. These approaches involved the same time domain pre-processing and differed in the focusing procedure.

The first processing was performed making use of the Reflexw commercial code and consisted of zero timing at 3 ns [24], background removal on all the traces [25], linear and exponential gain (with input parameters 1 and 1.2, respectively) and Butterworth filtering in the band of 300–1700 MHz. After this pre-processing, Kirchoff migration [16] was performed, extending the summations on 50 traces and exploiting a propagation velocity of 13 cm/ns. The value of the propagation velocity of the waves in the soil was retrieved from the diffraction hyperbolas of the pipes.

The second processing involved the same pre-processing step listed above, but the focusing was achieved by means of a linear inverse scattering algorithm based on the Born Approximation [19,26,27]. In particular, the inverse scattering problem was solved in a regularized way thanks to the truncated singular value decomposition [28] of the operator relating the pre-processed data to the unknown dielectric contrast, which is the objective function accounting for the targets to be retrieved. The inverse scattering algorithm worked in the frequency domain (so a Fourier Transform of the data is implicit) and was applied by means of a homemade code implemented in the MATLAB 7 environment. Moreover, it is intrinsically and computationally more demanding than the migration because the focus passes through the numerical computation of the discretized scattering operator and its singular value decomposition. Herein, data in the 450–1350 MHz band were exploited with a frequency step at 50 MHz, and the regularized solution was obtained by retaining only the singular values (and the relative singular functions) not smaller than 25 dB with respect to the first (maximum) one. The choice of the threshold was heuristic, i.e., based on the visualization of the results achieved for different trial values of the threshold.

For both approaches (migration and linear inverse scattering), the customary time–depth conversion (according to the propagation velocity of the waves in the soil) and the combined time–depth conversion have been applied. The combined time–depth conversion was implemented through a homemade code written in MATLAB, whereas the slicing was implemented using a dedicated Reflexw routine.

## 5. Results

This section presents some results achieved by using the migration-based processing and then those referring to the linear inverse scattering approach. 

### 5.1. Migration-Based Results

**Figure 4:**
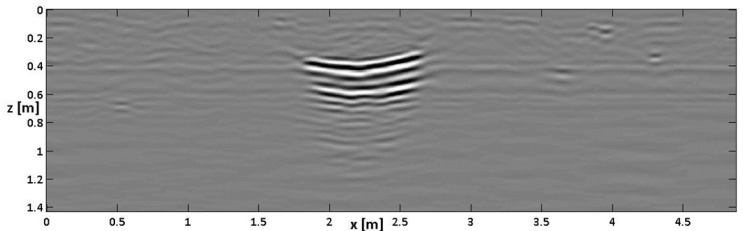
The result of a standard processing on Bscan no. 6.

Figure 4 shows Bscan no. 6 pre-processed (out of the 18 gathered Bscans, according to the scheme of Figure 3), migrated, and time–depth-converted according to the propagation velocity of the waves evaluated for the embedding medium. This Bscan crosses the buried box about in the middle. Abscissa and depth are represented with the same proportions to provide the natural aspect ratio of the retrieved buried scenario. 

The top and bottom of the cavity are both visible, and an inversion of the polarity of the reflection from the top and the bottom can be noted. In fact, a colorimetric sequence of white-black-white describes the top of the box, whereas a sequence of black-white-black describes the bottom. The top of the box appears slightly bent, likely because the box (initially flat) bent slightly under the weight of the overlying sand, especially when it was saturated with water and consequently handled more weight. Apart from this detail, we can read from Figure 4 an apparent cavity thickness of about 20 cm, whereas, as said, the real value is about twice larger. 

**Figure 5:**
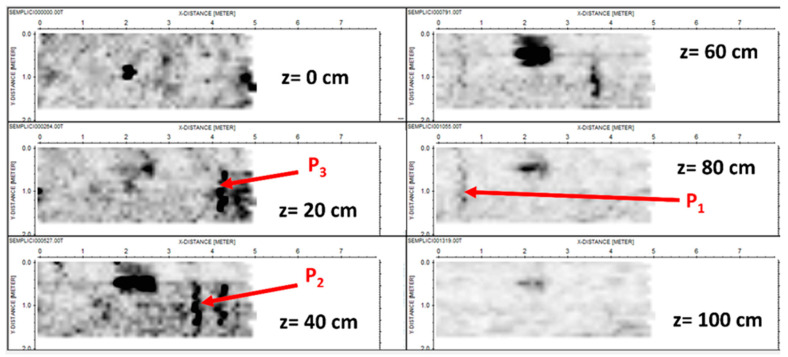
Standard depth slices. The units on the axes are in meters.

The depth slices with a depth step of 20 cm are shown in Figure 5. 

We can recognize the top and bottom of the box (at the apparent depths of 40 and 60 cm) plus three pipes, even if the deepest one, P_1_, appears to be weaker than the other two because the stronger attenuation of the signal (not fully counteracted by the gain vs. depth applied in the processing phase). Moreover, we can observe that the clearest echo of the pipe appears at 80 cm rather than its actual depth of 60 cm. This is likely because the echo of the pipe at 60 cm is partially covered by the strong echo from the cavity, apparently located at 60 cm in its turn. So, the residual part of the echo of the pipe appears more evident than the main echo. Moreover, we also see a meaningful superposition of the echoes of the other pipes on the left-right side of the cavity, especially at a depth of 40 cm. Finally, the central anomaly visible in the image at 0 cm (upper left panel) is associated with a piezometer buried in that point, namely a vertical pipe rising. This target is permanently present in the testbed, not removable, and is not represented in the scheme shown in Figure 3.

**Figure 6:**
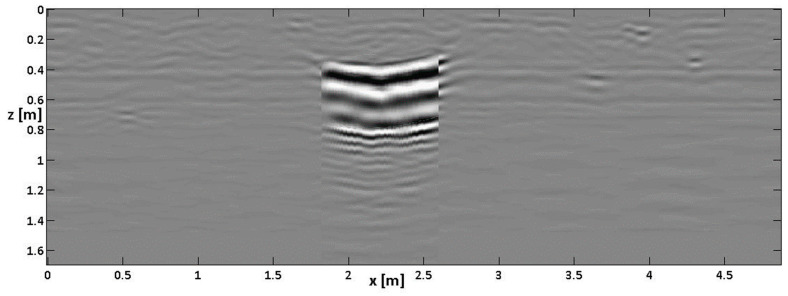
The result of standard processing with CTDC on Bscan no. 6.

The homologous image of Figure 4 after a CTDC is shown in Figure 6.

As can be seen, after the CTDC, the thickness of the cavity appears to be much closer to its actual value of 40 cm. A slight prolongation in Figure 6 of the spatial bottom scale of the image can also be appreciated with respect to Figure 4, which is coherent with the scheme of Figure 1. The zero padding at the bottom of the image is hardly visible in Figure 6 because it occurs at depth levels where the non-zero-padded part of the signal is strongly attenuated. 

**Figure 7:**
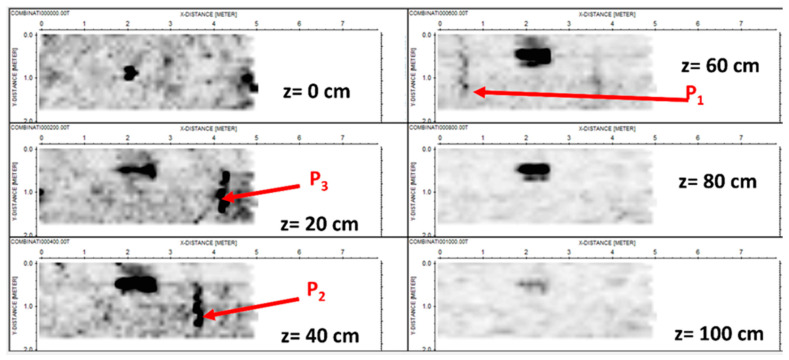
Depth slices achieved after combined time–depth conversion (CTDC). The units on the axes are in meters.

Figure 7 shows the slices achieved from the data after the CTDC. Indeed, the combined time–depth conversion has been applied only on the Bscans no. 5, 6, 7 and 8, namely the Bscans crossing the cavity. However, to have coherent files, a final zero padding has also been applied on the other Bscans, so we have built matrixes associated with the different Bscans with the same number of rows, which are needed to assemble the slices in Reflexw. 

From Figure 7, one can appreciate that the relationships between the depths of the buried targets are better respected. The depth of pipe P_3_ is correctly imaged, and the thickness of the cavity is better represented, with three “large” spots corresponding to them clearly visible in the spatial range from 40 to 80 cm. A first partial echo is visible also at 20 cm, partially due to the depth-averaging intrinsic in the slicing procedure [29] and partially because the top of the cavity was originally buried at 35 cm and not 40 cm. Figure 7 also shows that the pipes on the right-hand side of the cavity namely pipes P_3_ and P_2_, are imaged more clearly than in Figure 5. In particular, at a depth of 40 cm, we do not have a residual image of P_3_ close to P_2_, which occurred in the case of slicing without CTDC (see left-bottom panel in Figure 5). So, in the case at hand, the larger separation of the echoes from the top and bottom of the cavity mitigates some “confusion” present in the slices without CTDC.

### 5.2. Linear Inverse Scattering-Based Results

**Figure 8:**
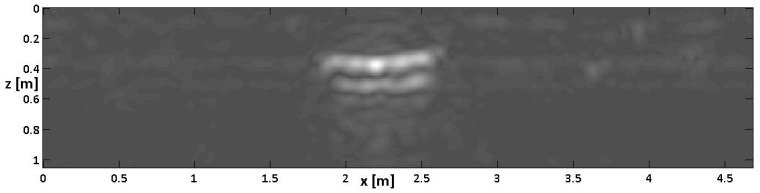
Reconstruction of Bscan no. 6 was achieved using a linear inverse scattering algorithm.

Figure 8 shows the result of the linear inverse scattering algorithm for the same Bscan from Figure 4. This time, the image represents the modulus of the contrast function, and therefore, no change of algebraic sign is present in the image of Figure 8. Indeed, the inverse scattering algorithm provides a result represented directly in the spatial domain. However, this does not elude the problem of the compression of the cavity because the algorithm accounts for the permittivity of the soil; it also accounts for the propagation velocity of the waves in the soil. In fact, as can be seen, the thickness of the cavity appears to be of the order of 20 cm in this case. 

**Figure 9:**
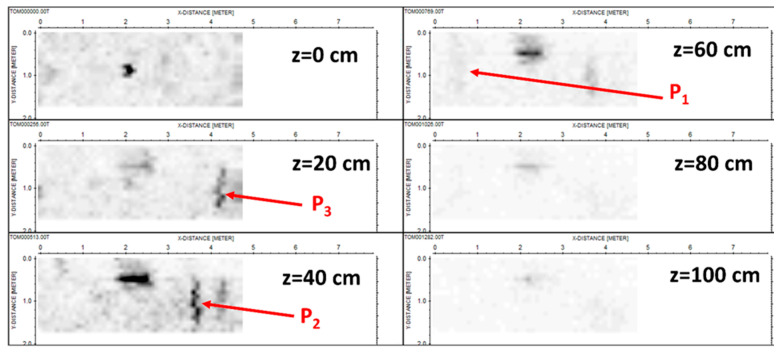
Depth slices were achieved from the tomographic reconstructions with standard slicing. The units on the axes are in meters.

Figure 9 (homologous to Figure 5) shows some horizontal slices achieved from the linear inverse scattering results. 

With regard to the slices achieved from the tomographic reconstruction, we might essentially repeat the same considerations exposed with regard to the slices achieved from standard processing based on the migration (see Figure 6). The only difference is that now pipe P_1_ is hardly visible.

**Figure 10:**
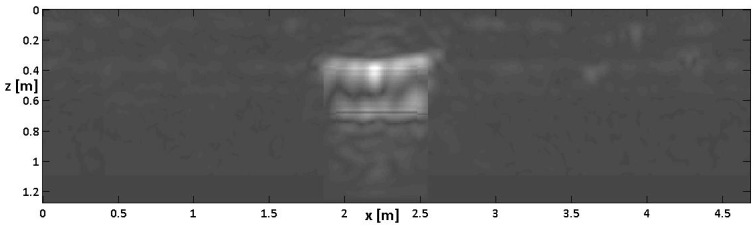
Reconstruction of Bscan no. 6 achieved from a linear inverse scattering algorithm with combined time–depth conversion (CTDC).

Figure 10 shows the same images as Figure 8 but are obtained using the CTDC. Indeed, as said in the case of an inverse scattering algorithm, we do not have a formal reconstruction “in the time domain”; thus, we do not have a formal time–depth conversion to implement. Notwithstanding, the essence of the problem does not change, and we achieve the correct cavity size by applying the same resampling of Equation (7) from the top to the bottom of the cavity. 

**Figure 11:**
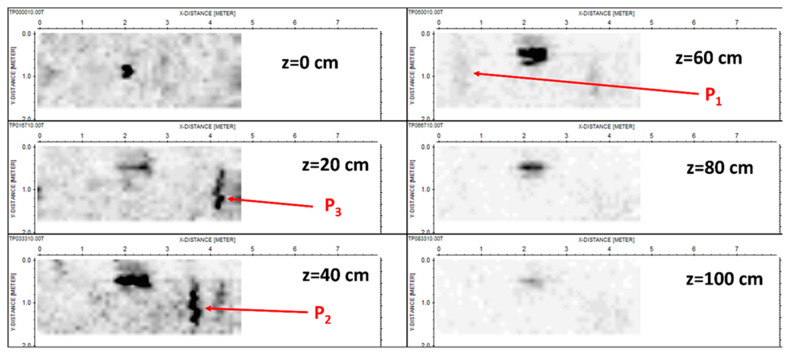
Depth slices built up with the reconstructions achieved from a linear inverse scattering algorithm after time–depth combined conversion (CTDC). The units on the axes are in meters.

Finally, Figure 11 (homologous to Figure 9) shows the slices achieved from the linear inverse scattering approach results and corrected by the CTDC. As for Figure 9, only Bscans no. 5, 6, 7, and 8 (i.e., those overflying the cavity) have been corrected with a CTDC. The others were just zero-passed at their bottom to have matrices of the same size for slicing.

In sum, in comparison with the homologous 5 and 7 without CTDC, Figure 9 and Figure 11 show that the extension of the cavity along the depth is better reproduced if a CTDC is applied. Moreover, the displacement along the depth of the three pipes is also better reproduced because the dynamic of each image results automatically better calibrated by the CTDC. Essentially, when the targets are not at the same apparent depth, it is less likely that the stronger reflections will cover the weaker ones. 

## 6. Conclusions

This paper has proposed a combined time–depth conversion (CTDC) for cavities embedded in the soil, accounting for the propagation velocity of the electromagnetic waves both in the soil and the cavity. This paper is part of a larger work [22,30] also investigating layered media (indeed, a cavity locally represents a particular three-layered medium, with upper and lower medium equal to the embedding soil and the central one constituted by free space). 

We have validated the approach vs. controlled the data gathered at the Hydrogeosite Laboratory of IMAA-CNR. After a common pre-processing, the data were focused on using a traditional migration algorithm working in a time domain and a linear inverse scattering approach working in a frequency domain. It is worth pointing out that the linear inverse scattering approach can be applied efficiently also in the case of electrically large-scale investigated domains, thanks to a technique called shifting zoom [31], which can dramatically reduce the computational burden without any meaningful loss of information. 

Regarding both data processing approaches, the results achieved without a CTDC show that the cavity appears meaningfully compressed, a well-known feature due to the different propagation velocities of the waves in and outside it. Conversely, even if the CTDC is based only on the modulus of the propagation velocity (it is evident that the CTDC does not consider the precise directions of the refracted waves), the experimental results show it allows for a meaningful correction of the thickness of the cavity.

Moreover, the CTDC is simple and not demanding regarding computational burden. Notwithstanding, the characterization of the top and the bottom of the cavities as two curves in the *x-t* plane may be time-consuming. In other words, the CTDC does not require any particular amount of CPU time but requires some time from a human operator.

For this reason, future research will focus on implementing a computer graphic algorithm, enabling the user to characterize buried interfaces by simply drawing them with the mouse. This will make the approach more user-friendly and less time-consuming.

## Data Availability

Data are contained within the article.
